# Killing the Invaders: NK Cell Impact in Tumors and Anti-Tumor Therapy

**DOI:** 10.3390/cancers13040595

**Published:** 2021-02-03

**Authors:** Martina Molgora, Victor S. Cortez, Marco Colonna

**Affiliations:** 1 Department of Pathology and Immunology, Washington University School of Medicine, St. Louis, MO 63110, USA; martina.molgora@wustl.edu; 2 Department of Medicine, University of California, San Francisco, CA 94143, USA; victor.cortez@ucsf.edu

**Keywords:** NK cells, innate lymphoid cells, ILC1, cancer, cancer therapy

## Abstract

**Simple Summary:**

NK cells are innate lymphoid cells involved in the control of tumor growth and metastatic spread. Given their significant cytolytic capacity, several promising strategies have been developed to target NK cells in cancer immunotherapy.

**Abstract:**

Natural Killer cells belong to group 1 innate lymphoid cells, which also includes ILC1s. NK/ILC1s are highly heterogeneous cell types showing distinct phenotypes across tissues and conditions. NK cells have long been described as innate lymphocytes able to directly and rapidly kill tumor cells without antigen-restriction. Different mechanisms were shown to modulate NK cell activation and tumor resistance, mainly based on cytokine stimulation and receptor–ligand interactions, and several strategies have been developed to target NK cells in tumor immunotherapy to promote NK cell function and overcome tumor evasion. The characterization of ILC1 distinct phenotype and function and the specific role in tumors still needs further investigation and will be essential to better understand the impact of innate lymphoid cells in tumors. Here, we review key aspects of NK cell biology that are relevant in tumor immune surveillance, emphasizing the most recent findings in the field. We describe the novel therapeutical strategies that have been developed in tumor immunotherapy targeting NK cells, and we summarize some recent findings related to NK cell/ILC1 transition in tumor models.

## 1. ILCs and Natural Killer Cells

Innate lymphoid cell (ILC) subgrouping mirrors the established paradigm of T cell subpopulations. Group 1 ILCs include conventional NK cells (cNK) and ILC1s. ILC1s resemble Th1 cells as they produce IFNγ and express the transcription factor Tbet. ILC1s are remarkably heterogeneous and display differential transcription factor dependencies and phenotypes, depending on the tissue. ILC1s share several similarities with cNK cells but are not as cytotoxic and do not express Eomes [1]. Recently, efforts have been made to characterize ILC1s features and core signatures and functions to distinguish them from conventional NK cells [2]. Given the diversity of ILC1 within and across tissues and conditions, it has been challenging to define a solid and conserved ILC1 transcriptional program in mice and humans. Moreover, it emerged recently that ILC1 subsets are not strictly tissue-resident populations but can actually migrate to different sites in both steady-state and inflammatory conditions. NK cells are innate lymphoid cells able to mediate resistance to damaged, stressed, virus-infected and tumor cells. NK cell activation relies on the equilibrium between activating and inhibitory signals, and the integration of these pathways prevents NK self-reactivity and promotes NK cell activation against cells in “distress” [3,4]. NK cells express a great variety of activating receptors that, upon interaction with specific ligands, drive NK cell triggering. NK cells, once activated, can release cytotoxic molecules, such as perforin and granzymes, and cytokines, such as IFNγ, thus participating in the shaping of the adaptive immune response [3,5,6].

## 2. The Impact of NK Cells on Tumor Immune Surveillance

NK cell impact on tumor immune surveillance in vivo was first reported around 1980, when genetic disorders causing an impaired NK cell function were associated with an increased tumor incidence in humans [7]. Accordingly, engineered mice with defective NK cell activity or mice depleted of NK cells were more susceptible to tumor growth and metastases [8,9]. Later, a pivotal epidemiological study showed that patients with reduced NK cell function were more prone to develop different types of cancer [10]. Additionally, NK cell deficiencies were correlated with increased tumor development, and NK cells were observed to be defective in oncological patients [11]. The limitation of these studies is that other immune cells and not only NK cells are affected by both the reported genetic defects in patients and by the depletion in murine models. NK cells have been reported to play a key role in the contexts of metastases and hematological malignancies. The actual contribution of NK cells in controlling solid tumors in patients still needs to be better defined, even though NK cells display killing capacities towards tumor cell lines of different origin in vitro. NK cells were proposed to be crucial for the early phases of tumorigenesis, even if an accurate demonstration of this concept is still challenging. Moreover, NK cells represent a small proportion of the immune infiltrate and NK cell association with prognostic factors is limited compared to CD8 T cells and regulatory T cells [12,13].

In the mouse, NK cell depletion experiments were supported by the observation that RAG-IL-2rγc-deficient mice (lacking B, T and NK cells) were more susceptible to tumors, compared to RAG-deficient mice (lacking B and T cells) [14,15,16]. IFNγ secretion and the consequent impact on macrophage phenotype was found to be responsible for NK cell-mediated tumor control in the methylcholanthrene (MCA)-induced sarcoma model.

## 3. NK Cell Recognition of Tumors

NK cell recognition of transformed cells is based on the balance of activating and inhibitory ligands expressed by target cells. MHC (major histocompatibility complex) class I are major inhibitory ligands. NK cells detect MHC class I through inhibitory receptors that include KIRs (in human), Ly49 (in mouse) and NKG2x/CD94 (in both mouse and human). Downregulation of MHC class I molecules in tumors, possibly because of a selective pressure mediated by CD8+ T cells, releases NK cells from inhibition, facilitating killing. NK cells can recognize and eliminate tumors that retain MHC class I expression but express one or several activating ligands, which engage activating receptors that overcome inhibitory receptors [12]. Here, we will focus on NK-cell-activating interactions. For a detailed review of inhibitory interactions, we refer to one of the excellent reviews on this topic (32800530; 33013909; 31231370). The best characterized activating receptors that recognize ligands expressed on tumor cells are NKG2D, CD226 (also known as DNAM-1) and NCRs, which include NKp46, NKp30 and NKp44 [17,18,19]. NKG2D recognizes class I-like molecules expressed on cells in distress and tumor cells, including MICA, MICB and ULBP in humans, as well as RAE1a-e, H60 and MULT1 in the mouse [20,21,22]. CD226 recognizes the Nectin-family ligands CD155 and CD112 on tumor cells [23]. Several ligands expressed by tumors have been proposed to engage the NCRs, including heparan sulfates, which engage NKp46 and NKp44, and B7-H6, which binds Nkp30 [24]. These receptor-ligand interactions have been examined in various tumor models. NKG2D-deficient (Klrk1^−/−^) mice are more susceptible to the development of several types of primary tumors, such as a transgene-driven lymphoma and prostate carcinoma, and the overexpression of NKG2D ligands is protective in models of transplanted tumors [14,21,25]. In vitro cytotoxicity assays using blocking antibodies first unveiled the contribution of NCRs in NK cell anti-tumor activity [18]. Nkp46-deficient (Ncr1^−/−^) mice were then shown to be more susceptible to different transplantable models (i.e., lymphoma, Lewis lung carcinoma, B16 melanoma metastasis and glioblastoma) [26,27,28].

One emerging aspect in NK cell biology and anti-tumor activity is the NK cell capacity to recognize growth factors that can profoundly impact tumor immune surveillance. It was recently reported that NKp44 binds to the extracellular matrix protein Nidogen-1 (NID1) and the Platelet-Derived Growth Factor (PDGF)-DD, leading to an inhibitory and activating signal, respectively [29,30]. NKp44 engagement by PDGF-D elicits IFNγ and TNFα production in NK cells and drives a transcriptional program associated with NK cell activation and survival. PDGF-D-mediated NK cell activation inhibits tumor growth in vitro and in vivo in a model of B16-induced lung metastasis in NKp44-transgenic mice [29]. PDGF-D is expressed by various human tumors, both by tumor cells and vascular endothelial cells in the tumor microenvironment. Its canonical signaling via PDGFRβ is associated with robust tumor growth and progression, vascular proliferation, angiogenesis, metastatization and stromal reaction [29,31,32]. On the other hand, PDGF-D sensing via NKp44 activates innate immune cells and promotes tumor control [29]. Importantly, in a TCGA cohort of glioblastoma (GBM) patients, a core signature of PDGF-D-induced genes in NK cells was positively correlated with NCR2 and PDGF-D/NKp44 engagement downstream events were associated with greater overall survival [29], suggesting a potential impact of NK cell activation in brain tumors.

The specific contribution of NK cells in primary tumor immune surveillance still needs to be better characterized, since NCRs, DNAM1 and NKG2D expression is shared with subsets of T cells, ILCs and NKT cells. For instance, it was reported that NKG2D-deficiency was associated with increased susceptibility to diethylnitrosamine (DEN)-induced hepatocellular carcinoma and NKG2D+ T cells were responsible for the phenotype [33].

## 4. NK Cell Subsets in Solid Tumors and Hematological Malignancies

In humans, blood NK cells encompass two major subsets: CD56^bright^CD16^–^ specialize in IFNγ secretion; CD56^dim^CD16^+^ are more cytotoxic. Intermediate subsets with diverse expression of CD56 and CD16 have been identified in several contexts, suggesting heterogeneity and phenotypic plasticity among the populations. NK cell subset distribution is significantly altered in cancer patients, and distinct NK cell subsets may play differential roles during tumor progression [34,35,36]. Less cytolytic CD56^bright^CD16^–^ NK cells were found to be enriched in solid tumors, such as breast, melanoma, colon and non-small cell lung cancer (Bruno et al., 2013; Levi et al., 2015). Tumor-infiltrating NK cells (TINK) displayed a different signature compared to circulating CD56^high^CD16^−^ NK cells and expressed increased levels of CD9 and CXCR3. TINKs were shown to be less cytolytic and produce VEGF, resembling decidual NK cells (dNK) and having a pro-angiogenic and tumor-promoting phenotype [36,37]. The accumulation of poorly cytotoxic NK cell subsets in tumors was reported to be dependent on the tumor chemokine milieu in breast and lung cancer. Chemokines responsible for the migration of CD56^bright^ NK cells (i.e., CXCL9 and CXCL10) were enriched, whereas chemokines responsible for the migration of CD56^dim^ NK cells (i.e., CXCL2) were reduced in cancer patients. Less mature and less functional NK cells (CD16^−^CD117^high^CD27^high^CD57^low^) were increased in both peripheral blood and tumor microenvironments in patients with advanced mammary tumors. Furthermore, defective expression of NK-cell-activating receptors was described in cancer patients [38,39]. For example, NK cells in prostate cancer patients showed a decreased level of activating receptors, such as NKp46, NKp30, DNAM1 and CD16, and an increased level of inhibitory receptors, such as ILT2. Interestingly, more marked alterations were observed in NK cell phenotype in metastases compared to primary lesions [40,41]. Compromised NK cell activity was also reported in hematological malignancies (acute and chronic leukemia, myelodysplastic syndromes and multiple myeloma). In particular, NCR^low^CD16^+^KIR^+^ NK cells were accumulated in acute myeloid leukemia patients and showed poor anti-tumor effector functions [41,42]. A CD56^+^ population displaying mixed features of conventional cytotoxic NK cells and ILC1s was recently described as impaired in acute myeloid leukemia (AML) patients [43]. These so-called CD56^+^ ILC1-like cells have strong cytolytic capacities that were shown to be KIR-independent and express high levels of TRAIL, NKp30, NKp80 and NKG2A. Their cytotoxic potential was dramatically reduced in AML patients possibly through HLA-E or TGFβ, and interestingly, it was restored upon remission [43]. In patients with myelodysplastic syndromes (MDS), AML and multiple myeloma (MM), the frequency of NKG2D^+^ and DNAM1^+^ NK cells was reduced and in acute lymphoid leukemia (ALL) and AML patients, and circulating NK cells expressed increased levels of NKG2A and decreased levels of NKp46 [44,45]. A novel subset of NK cells (CD56^low^CD16^low^NKG2A^+^) was identified in pediatric ALL patients. This subset had high cytolytic potential and produced high levels of IFNγ in healthy donors, but it was functionally defective in ALL patients [46]. Clinical studies have shown that CMV reactivation in transplanted patients drove the expansion of NKG2C^+^CD57^+^ NK cells that were associated with lower relapse rates, indicating a beneficial role of this population in cancer patients [47,48]. In chronic leukemia patients, the NK cell subsets proportions and phenotype were comparable to healthy individuals, but the cytolytic capacity was shown to be impaired [49].

NK functionality in tumors can be impaired in different ways, including the regulation of NK cell maturation and NK cell subsets recruitment, NK cell receptor modulation and the direct suppression of NK cell functions. The characterization of the diverse mechanisms that contribute to NK cell functional defects in different tumors may pave the way to the development of novel therapeutical strategies to selectively target NK cells in different contexts.

## 5. NK Cell Anti-Metastatic Activity

Even though the contribution of NK cells in controlling primary solid tumors is still a matter of debate, their importance in the regulation of metastatization has been largely described [50]. For instance, in numerous types of tumors (e.g., gastrointestinal sarcoma, gastric, colorectal, renal and prostate carcinoma), NK cell frequency in the periphery or in the tumor was inversely correlated with the metastatic spread, which is one of the major causes of cancer-associated death (Delahaye et al., 2011). In several cohorts of patients with metastatic tumors or at risk of metastases, NK cell activation correlated with a favorable prognosis. For example, NKG2D and NKp46 expression in tumor-infiltrating NK cells correlated with reduced metastatization after surgery in patients with prostate cancer [51]. In several transplantable tumor models in the mouse, NK cell depletion led to reduced control of metastatization and did not influence primary tumor growth. The impact of NK cell deficiency on the metastatization process was observed in tumor-bearing mice injected via different routes (intravenous, intracardiac, subcutaneous, intrasplenic or orthotopic) [21,52,53]. In agreement, conditions and treatments that promoted NK cell function had a beneficial effect in metastasis models [54,55,56]. Molecules that regulate NK cell response to cytokines were described as potent modulators of NK cell activity. IL-1R8 is an IL-1 family member that negatively controls IL-18 signaling in NK cells, and IL-1R8 genetic blockade was shown to promote NK cell function in models of metastasis and liver primary tumors [57]. CIS inhibits IL-15 signaling, and CIS-deficient NK cells promoted resistance to melanoma, prostate and breast cancer metastases in vivo [54]. Recently, it was observed that the Dectin-1/MS4a4a axis in tumor-associated macrophages is associated with better control of lung metastases, which was shown to be dependent on NK cells [58].

The mechanisms by which NK cells are more capable of eradicating metastases than tumors at the primary site still need to be clarified. Epithelial to mesenchymal transition (EMT), which is a hallmark of the metastatization process, was shown to induce an increased expression of NKG2D ligands and a reduction in the inhibitory ligands, thereby promoting NK cell killing [59,60]. Moreover, if the major contribution of NK cells in controlling metastases occurs at the primary site, in the circulation or in the secondary site is still controversial. It is known that NK cell recruitment and tissue-specific enrichment are key steps of NK cell anti-metastatic activity, but the exact mechanism needs to be elucidated [50,61]. One of the major limitations in the field is that most preclinical models rely on tumor cell line injection; therefore, lacking the early stages of metastatization. For the same reason, a comprehensive analysis of NK cell impact on solid tumors may be challenging because of the technical difficulty in generating models to analyze the initial stages of tumorigenesis.

## 6. Tumor Immunoediting and Evasion of NK Cell Recognition

MCA-derived tumors induced in mice lacking NK cells were observed to be more immunogenic upon transplant in immunocompetent wild-type hosts, suggesting a contribution of NK cells in the tumor immunoediting process [16]. In line with this, MCA-derived sarcomas and DMBA-derived papillomas originated in DNAM1-deficient (Cd226^−/−^) mice were shown to express higher levels of the DNAM1-activating ligand CD155. Sarcomas isolated from NKp46-deficient mice (Ncr1^−/−^) expressed higher levels of NKp46 ligands [15]. NKG2D-mediated immunoediting was also reported in MCA-sarcoma models from mice lacking perforin (Prf1^−/−^) and in B cell tumors from NKG2D-deficient mice (Klrk1^−/−^) (Guerra et al., 2008; Iguchi-Manaka et al., 2008; Takeda et al., 2005).

Tumors have established numerous strategies to escape immune surveillance, including mechanisms to evade the NK cell-mediated response. It was observed that NKG2D ligands (NKG2DLs) could be cleaved, leading to a reduced NKG2D engagement and NK cell activation. In line with this, soluble forms of NKG2DLs were found in the serum of cancer patients, indicating a potential diagnostic implication of NKG2DL detection [62,63,64,65]. Moreover, tumor cells can induce the expression of NKG2DLs in host myeloid cells via soluble factors such as lactate dehydrogenase [66]. The treatment with a recombinant NKG2D ligand (MULT-1) was shown to rescue the de-sensitization of NK cells driven by NKG2DL shedding and NKG2D binding by myeloid cells [67]. The NKp30 ligand B7-H6 was reported to be shed by tumor cells through metalloprotease-mediated cleavage, and high levels of soluble B7-H6 were found in the serum of patients with melanoma, suggesting a B7-H6-decoy mechanism of immune evasion [68]. Additionally, other ligands of NK cell receptors (i.e., CD48 that binds 2B4, PVR that binds DNAM-1 and CD58 that binds CD2) have been detected in human serum and may function as decoys that impair receptor engagement in NK cells [69,70,71].

Additional evasion mechanisms developed by tumor cells comprise the expression of suppressive molecules that inhibit NK cell function (e.g., TGFβ, prostaglandin E2 (PGE2), indoleamine 2,3-dioxygenase (IDO), adenosine and IL-10), the expression of ligands that engage NK cell inhibitory receptors and the activation of platelets that reduces NK cell activation [72,73,74,75,76].

Hypoxic conditions commonly occur in solid tumors, influencing immune cell phenotype and functions [77]. Recently, it was observed that HIF-1α upregulation induced in tumor-infiltrating NK cells is detrimental to NK cell anti-tumor capacities [78]. Indeed, Hif1a specific deletion in NK cells drives tumor rejection in NK cell-sensitive solid tumor models, and it is associated with increased NK cell activation in terms of IFNγ production and cytoxicity. NK cell phenotype in the absence of HIF-1α was dependent on IL-18, and the enrichment of NK-IL-18-IFNG signature correlated with better prognosis in patients with solid tumors [78].

Finally, the tumor microenvironment has an impact on the expression pattern of NK cell receptors, inducing alterations that promote a less reactive and less cytolytic phenotype of NK cells, as described above (Section 4).

## 7. Tumors, TGFβ and Transition: NK Cell-ILC1 Identity

**Figure 1:**
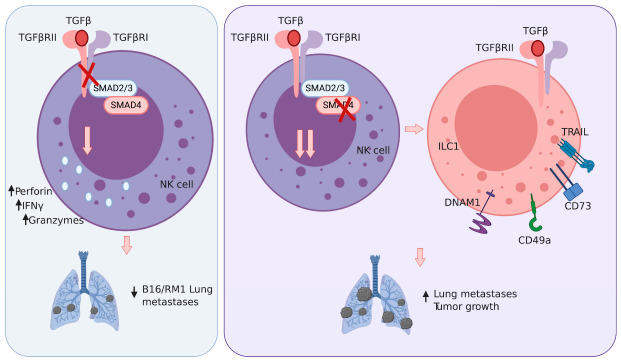
Impact of TGFβ signaling in NK cells/ILC1s in tumors. Left panel: TGFβ signaling blockade promotes NK cell activation (perforin, granzymes, IFNγ production) in vitro and lung metastases control in vivo. Right panel: SMAD4 genetic blockade results in a more pronounced ILC1 phenotype, and this is associated with reduced tumor control in vivo. Whether this is due to a reduced NK cell anti-tumor activity or a more prominent ILC1 phenotype is still to be defined.

TGFβ has a well-established role as a cytokine in the tumor microenvironment that is strongly immunosuppressive [79]. TGFβ signaling is initiated by binding of the cytokine to its cognate receptors (TGFβR-II and TGFβR-I), which facilitates phosphorylation of the SMAD transcription factors (Smad2/3). These receptor SMADS then associate with Smad4, and this complex translocates to the nucleus to initiate gene transcription. NK cells express TGFβ-RII in the steady state; however, NK cell-specific deletion of this receptor (Tgfbr2ff × Ncr1Cre) does not result in a notable phenotype under homeostatic conditions [80,81] (Figure 1). Interestingly, CD11c driven expression of a dominant negative TGFβ-RII did result in altered NK cell maturation in the steady state [82]. The differences in these two models may be due to the stage of NK cell development in which NKp46 and CD11c are expressed, and thus TGFβ signaling is abolished.

In the context of cancer, TGFβ has been shown to suppress NK cell-mediated anti-tumor responses. Culture of NK cells with TGFβ impairs their functional capabilities, including reduced IFNγ, granzyme and perforin production as well as decreased cytolytic degranulation [80]. In vivo, Tgfbr2ff x Ncr1Cre mice were better protected from B16F10 and RM1 lung metastasis compared to their wild-type counterparts [80] (Figure 1). This protection was reversed upon antibody-mediated depletion of NK cells, demonstrating that TGFβ signaling can impact NK cell control of metastasis. While the ability of TGFβ to inhibit NK cell anti-tumor function is in line with this cytokine impact on other immune cells, TGFβ can also directly promote the conversion of NK cells into ILC1-like cells within a tumor environment.

TGFβ has a unique role in controlling the cellular identities of NK cells and ILC1. The in vitro treatment of NK cells with TGFβ induces the expression of multiple genes associated with ILC1 identity, including: CD49a, TRAIL, DNAM-1 and the downregulation of Eomes [81,83,84]. Conversely, ILC1 within the salivary glands require TGFβ to maintain their expression of ILC1 signature genes, and when TGFβ signaling has been abolished, they begin to resemble NK cells [81]. Intriguingly, examination of group 1 ILC infiltrates in fibrosarcoma tumors (MCA 1956) revealed three distinct populations, identified as NK cells (CD49a-CD49b+), intermediate ILC1 (CD49a+CD49b+) and ILC1 (CD49a+CD49b-) [84]. All three populations were shown to arise from NK cells, which upon entering the tumor microenvironment acquired these distinctive gene expression profiles. This conversion of NK cells into ILC1-like cells is dependent on TGFβ signaling, as the intratumoral intermediate ILC1 and ILC1 populations were strongly reduced in Tgfbr2ff x Ncr1Cre mice. These results demonstrate that the ability of TGFβ to drive the conversion of NK cells to an ILC1-like identity can occur in vivo within the tumor microenvironment [84].

Currently, it is unclear if the TGFβ-mediated conversion of NK cells to ILC1-like cells results in poorer control of tumor growth. While Tgfbr2ff x Ncr1Cre mice are better able to control lung metastasis and solid tumor growth, it is difficult to distinguish whether this is simply due to enhanced cytotoxic capabilities of NK cells or is directly related to the inability of NK cells to convert to ILC1-like cells. Two mouse models have been reported in which NK cells appear with an ILC1-like phenotype under homeostatic conditions (Smad4ff x Ncr1Cre mice and Mir142^−/−^ mice) and confirm that this phenotype is associated with poorer cytotoxic function in vitro and tumor control in vivo [85,86,87]. ILC1 and ILC1-like cells do express several molecules that have been shown to inhibit T cell immunity, such as TRAIL and CD73, suggesting that these cells may directly suppress anti-tumor immune responses [88,89]. However, some tumor types may be sensitive to TRAIL-mediated killing, and in this scenario, ILC1 and ILC1-like cells would help control tumor growth. Clearly, an outstanding question for future work is whether the TGFβ mediated conversion of NK cells to ILC1-like cells within the tumor microenvironment has a beneficial or detrimental impact on cancer growth.

## 8. NK Cell-Based Immunotherapy Approaches

**Figure 2:**
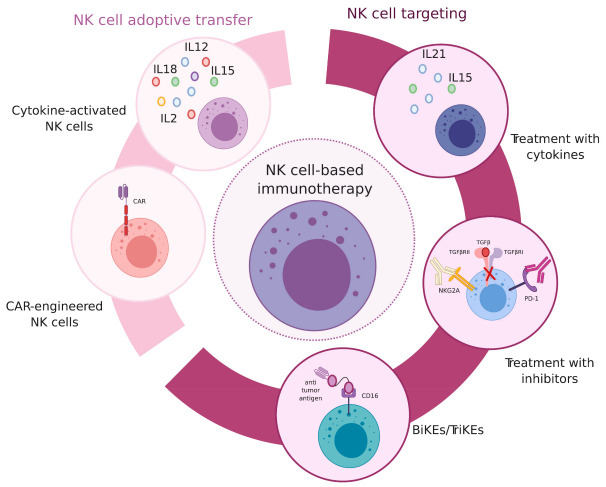
NK cell-based immunotherapy strategies. Different therapeutical approaches that were recently developed to enhance NK cell anti-tumor potential. From the right part, treatments to promote NK cell function: treatment with cytokines to promote NK cell activation; treatment with antibodies or inhibitors to block NK cell inhibitory receptors or inhibitory pathways; NK cell engagement via the bi/tri-specific molecules, simultaneously targeting NK cells and tumor antigens to link activating receptors in NK cells and tumors. From the left part, NK cell therapy: NK cell adoptive transfer, upon pre-treatment with cytokines; infusion with CAR-NK cells.

The development of novel immunotherapy strategies has been a breakthrough in oncological treatments, and the recovery or enhancement of NK cell antitumor capacity is one of the targets of several clinical trials in cancer patients. The emerging literature further characterizing NK cell regulation, crosstalk with other cell types in the tumor microenvironment, trafficking and localization, heterogeneity and plasticity has been crucial to gain insight into NK cell antitumor potential and to establish novel therapeutical approaches [90] (Figure 2).

### 8.1. NK Cell Therapy

The first cell therapy approach using NK cells was established more than 30 years ago, and it consists of the infusion of NK cells activated with IL-2, named lymphokine-activated killer (LAK) cells [7]. The initial evidence of a clinical benefit due to NK cell transplant is the allogeneic bone marrow transplantation in AML patients with an HLA-C mismatch [91]. Mismatched alloreactive NK cells were reported to acquire a better capacity to eradicate blasts that cannot properly engage the autologous pattern of KIRs. The NK cell response to leukemia was therefore more efficient in the allogeneic setting and did not cause graft-versus-host-disease (GVHD) [91].

Recently, several strategies based on the adoptive cell therapy of NK cells have been tested, and the source of primary NK cells can be either autologous or allogeneic [92]. In pediatric AML patients, it was shown that haploidentical NK cell transplant led to complete remission and disease-free survival [91,93,94]. Further studies revealed that treatments based on NK cell KIR mismatch did not influence the response to hematological tumors other than AML, and autologous NK cell transfer alone was not protective in patients with different types of solid tumors (renal-cell carcinoma, advanced gastrointestinal cancer and metastatic melanoma) [91,95,96]. The impact of NK cells in hematological malignancies in pediatric tumors has been extensively reviewed elsewhere [97,98].

NK cell pre-activation with the combination of different cytokines (i.e., IL-2, IL-12, IL-15, IL-18, IL-21) was used to boost NK cell function, and it was observed that the transfer of IL-15-treated NK cells was associated with a better response in solid tumors in a pediatric cohort [99,100]. Clinical trials have been examining the impact of NK cell infusion in combination with IL-15 in solid and hematological tumors and anti-CD19 CAR-engineered NK cells in leukemia patients [101]. CAR-NK cells represent a promising immunotherapy strategy and might show advantages compared to CAR-T cells, such as the induction of less severe side effects and an easier manufacturing process [101,102]. In this regard, it was recently shown that CAR-NK cells do not cause any relevant cytokine storm in patients with lymphoid tumors, although the adverse effects related to NK cell treatments still need to be investigated in other conditions [102,103]. Several clinical trials are ongoing to test the usage of cytokine-induced memory-like NK cells (CIML-NK) that are generated by ex vivo treatment of allogeneic cells with a combination of cytokines (e.g., IL-12, IL-15 and IL-18) [104]. They were shown to have a phenotype distinct from conventional NK cells, improve effector functions and induce remission in AML patients [104,105].

Various protocols for the in vitro manipulation and infusion of NK cells have been tested and significantly improved, even though the cryopreservation and engineering of primary NK cells still represents a challenge and a limitation for clinical applications.

### 8.2. Targeting NK Cell Inhibitory and Activating Pathways

NK cell targeting in cancer therapy can rely on the blockade of inhibitory receptors or, on the other hand, on the engagement of activating pathways, including cytokine receptors. The first NK cell-targeting treatment was IL-2 injection, which indeed promoted NK cell activation in patients but also had severe side effects including vascular leakage and organ injury [7,106]. Moreover, IL-2 induces NK-cell but also T-regulatory-cell proliferation and activation and therefore immunosuppression. For these reasons, variant forms of recombinant IL-2 were generated in order to gain a higher affinity to the NK cell IL2R chains and lower affinity for the IL2Rα chain present on Tregs [107]. Treatment with recombinant IL-15 or IL-15-IL15Rα complexes is being tested in metastatic patients in clinical trials [108]. A protocol based on the sequential use of IL-15 and IL-21 to enhance NK cell proliferation and cytotoxicity has been also established [109]. TGF-β is one of the major suppressive cytokines acting on NK cells, and the inhibition of TGF-β pathway has been tested in cancer patients using the pharmacological inhibitor (galunisertib). NK cells can also exert ADCC and therefore impact the efficacy of several antibody therapies. Moreover, bispecific or trispecific killer-cell engagers (BiKE or TriKE) binding CD16 and tumor antigens were generated to trigger CD16 and promote the consequent ADCC response [110,111]. BiKEs engaging tumor antigens and the activating receptor NKG2D were also developed and tested in murine models of multiple myeloma [112]. Recently, a tri-functional NK cell engager (NKCE) that triggers both NKp46 and CD16 on NK cells and simultaneously a tumor antigen (e.g., CD19, CD20 and EGFR) has been developed. NKCEs showed enhanced anti-tumor activity in preclinical models compared to the mAb treatments alone (e.g., rituximab, obinitumab, cetuximab) [113].

PD-1 blockade has been a revolution in oncological treatments, and it emerged that PD-1 can be expressed in activated NK cells, in certain conditions [114,115,116]. Immune checkpoint therapy (ICT) targeting PD-1 was shown to have an effect on NK cell activation in multiple myeloma patients and the murine model [115,117,118,119]. Anti-PDL1 treatment promoted NK cell-mediated ADCC against tumor cells (Boyerinas et al., 2015). Additionally, Tim3 blockade was associated with an increased NK cell activation in melanoma patients [120]. The impact of CTLA-4 blockade on NK cells still needs to be elucidated and may be an indirect effect of T cell reactivation [121,122]. The blockade of NK cell inhibitory receptors has been tested in clinical trials. In multiple myeloma patients, KIR2DL-1, KIR2DL-2 and KIR2DL-3 targeting did not show any beneficial effect as a single agent and the combinations with other treatment are currently under investigation in patients with hematological malignancies and solid tumors [123,124,125]. Monalizumab has been recently generated, and it is a monoclonal antibody specifically recognizing and blocking NKG2A. There are several ongoing trials to test the impact of NKG2A blockade in different types of tumors, even if the effect may be dependent on both T cells and NK cells [126,127].

A better characterization of NK cell responses in different types of tumors will be important to improve current immunotherapy approaches, to identify novel targets and to develop specific therapeutical strategies.

## 9. Concluding Remarks and Future Perspectives

Various receptor–ligand interactions and pathways have been described over the years in the context of NK-cell-mediated tumor surveillance. Most of them have been characterized as important players in tumor control by modulating NK cell phenotype and functions. Several therapeutical strategies were consequently developed based on the engagement of the activating and/or blockade of the inhibitory signals to enhance NK cell activation and tumor cell killing. It would now be crucial to establish approaches to define and stratify patients and tumor types that might specifically benefit from one particular treatment or a combination of treatments. That might be achieved with the assessment or prediction of receptor-ligand interactions enriched in different tumors.

Regarding NK cell/ILC1 distinction and characterization, the identification of specific core genes and differential functions of the two populations has been one of the major focuses of this field. Given the significant heterogeneity of NK cells and ILC1s not only in different pathological contexts but also among different compartments and tissues at the steady-state level, it remains challenging to define differential phenotypes and functions. Historically, NK cells have been described as cytotoxic innate lymphocytes, whereas ILC1s are supposed to be less (or not) cytotoxic and specialized in cytokine production. This concept is based on the differential expression of cytotoxic mediators (i.e., granzymes and perforin) and the lack of the transcription factor Eomes in ILC1s. Given the high expression of TRAIL in ILC1s, cytotoxic mechanisms cannot be excluded and might be limited to certain conditions depending on TRAIL receptor expression.

Finally, ILC1s were first defined as a tissue-resident population, whereas NK cells are found both in the circulation and in tissues. Recently, it emerged that ILC1s can actually migrate in certain conditions. For example, it was shown that liver ILC1s can be sensitized in draining lymph nodes and traffic back to the liver. Further research to gain a greater genetic and molecular definition of NK/ILC1 cell identity will help our understanding of their function and pave the way for novel therapeutical strategies.
